# Interspecific hybridization for transfer of hull-less seed trait from *Cucurbita pepo* to *C. moschata*

**DOI:** 10.1038/s41598-023-29935-9

**Published:** 2023-03-21

**Authors:** Barinder Kaur, Karmvir Singh Garcha, Jagdeep Singh Sandhu, Madhu Sharma, Ajmer Singh Dhatt

**Affiliations:** 1 Department of Vegetable Science, Punjab Agricultural University, Ludhiana, 141004 India; 2 School of Agricultural Biotechnology, Punjab Agricultural University, Ludhiana, 141004 India; 3 Directorate of Research, Punjab Agricultural University, Ludhiana, 141004 India

**Keywords:** Plant sciences, Plant breeding

## Abstract

Hull-less seed trait is preferred by nut and oil industries worldwide for snacking and oil extraction as it evades the expensive decorticating (dehulling) process. This seed trait is available in *C. pepo* only, which has small seed cavity, sensitive to various biotic and abiotic stresses, and restricted to temperate regions for cultivation. Contrarily, the related species *C. moschata* has wider adaptability, disease tolerance and high seed yield. Therefore, attempt was made to transfer this trait into *C. moschata* through conventional pollination and ovule culture using four parents of hull-less *C. pepo* and six of hulled *C. moschata*. Through conventional approach, few viable F_1_ seeds (12–23) were obtained by using *C. pepo* as female parent, but in three crosses (HLP36 × HM1343, HLP36 × HM1022 and HLP44 × HM1022) only, whereas, its use as male parent was not successful. This incompatibility issue of reciprocals was resolved through ovule culture of *C. moschata* genotypes HM1343 and HM6711 after 17 to 19 days of pollination with *C. pepo* genotypes HLP53 and HLP72, respectively. The hybridity of interspecific crosses was confirmed through SSR markers (alleles inherited from both the parents), morphological characters and micromorphological leaf traits (differed from both the parents). The successful transfer through interspecific hybridization was further established with the presence of hull-less seed in fruits of F_2_ populations. Outcome of this study would pave the way for enhancing the productivity and multi-season cultivation of snack-seeded pumpkin even in subtropical and tropical regions.

## Introduction

Genus *Cucurbita* includes 27 species, among them *C. pepo* L. and *C. moschata* D. commonly called squashes and pumpkin have been domesticated worldwide for their use as fruits and seeds^1^. The seeds are rich source of nutrients, oil and health enhancing fatty acids, which are considered to play role in prevention of prostate hyperplasia, maintaining hormonal balance, brain functions and skin wellbeing^2^. Pumpkin seeds are extensively used in bakery products, as snacks and for premium quality vegetable oil. Prior to its use, thick and leathery seed coat (hull) has to be removed, whereas use of hull-less seeded mutant can evade the labour intensive decorticating process. This mutant was emerged in Austria during 1880s and now being used extensively in Europe and North-American countries^3^.

We introduced hull-less seeded variety ‘Lady Godiva’ (*C. pepo*) from USA during 2009 and transferred this trait into local germplasm of *C. pepo* for release of ‘*PAU Magaz Kadoo-1*’, the first snack seeded variety of India^4^. *C. pepo* has the limitations of short growing season (February to May), narrow adaptability, high temperature sensitivity and virus susceptibility (zucchini yellow mosaic virus, cucumber mosaic virus and watermelon mosaic virus), whereas, a related species *C. moschata* has broad window of cultivation (February to November), wider adaptability, high temperature tolerance, virus resistance and better seed production potential^5,6^. Hence, transfer of hull-less seed trait from *C. pepo* to *C. moschata* can pave the way to solve above-mentioned issues being faced in sub-tropical and tropical regions.

Both of these species belongs to same family and have identical chromosome number (2n = 2x = 40), but hybridization among them is rarely successful due to cross-compatibility issues caused by both pre and post fertilization barriers^7–9^. Pre-fertilization barriers comprises low pollen viability, failure of pollen germination, variations in pollen tube and flower style length among the genotypes while post fertilization barriers include embryo or endosperm degeneration^9,10^. Attempts have been made to develop interspecific hybrids in the genus *Cucurbita* using conventional pollinations involving large number of diverse genotypes and/or embryo rescue approach with limited success^10–12^.

Success in interspecific hybridization among *Cucurbita* species through conventional approach has been achieved by transferring bush growth habit from *C. pepo* to *C. moschata*^13^, cucumber mosaic virus (CMV) resistance from *C. moschata* to *C. pepo*^14^, zucchini yellow mosaic virus resistance from *C. moschata* to *C. pepo*^15^ and powdery mildew resistance from *C. okeechobeensis* to *C. moschata* and *C. pepo*^16^. Embryo rescue is an in vitro culture techniques used to assist the development of immature or weak embryos that might not survive to become viable plants. It has been used for developing interspecific hybrids between *C. moschata*, *C. pepo, C. ficifolia* and *C. martinezii* to widen the genetic base^17^, *C. pepo* and *C. moschata* for papaya ring spot virus resistance^18^ and *C. martinezii* and *C. pepo* for powdery mildew and cucumber mosaic virus resistance^19^.

Therefore, present study was planned to transfer hull-less seed trait from *C. pepo* to *C. moschata* through conventional pollination and embryo rescue approaches with the anticipation to have hull-less seed trait in high yielding, virus resistant and wider adaptable genotypes of the pumpkin. This study highlights (i) hybridization through conventional pollination and embryo rescue approach (ii) confirmation of putative hybrids using molecular, morphological and micromorphological observations (iii) characterization of segregating (F_2_) populations for hull-less seed trait derived from interspecific crosses.

## Results

### Interspecific hybridization

Total 916 pollinations of 48 cross combinations were attempted involving four parents of *C. pepo* and six of *C. moschata* in reciprocal manner (Table 1). In *C. pepo* × *C. moschata* crosses, fruit setting was observed in eight combinations with the highest setting in HLP36 × HM1343 followed by HLP36 × HM1022 and HLP44 × HM1022 having 15, 23 and 12 number of developed seeds per fruit, respectively. Out of which 6.79, 10.20 and 5.50 seeds were germinated with 3.35, 4.8 and 2.5 number of survived seedlings per fruit from the respective crosses. In reciprocals i.e. *C. moschata* × *C. pepo,* seed was obtained from two crosses (HM1343 × HLP53 and HM6711 × HLP72) only, but failed to germinate.

**Table 1:** Interspecific hybridization between *C. pepo* × *C. moschata* and their reciprocal crosses through conventional pollination.

Cross combination		Number of pollinated flowers	Fruit set		Number of seeds per fruit	Number of seeds germinated per fruit	Number of seedlings survived per fruit	Total number of plants acclimated
Female parent	Male parent		Number	(%)				
*C. pepo*	*C. moschata*							
HLP36	HM1404	17	1	5.88	0	–	–	–
	HM108	22	1	4.54	0	–	–	–
	HM1343	21	14	66.67	15	6.79	3.36	47
	HM1022	22	5	22.73	23	10.20	4.80	24
	HM2211	16	0	0	–	–	–	–
	HM6711	19	0	0	–	–	–	–
HLP44	HM1404	16	0	0	–	–	–	–
	HM108	18	1	5.55	0	–	–	–
	HM1343	21	1	4.76	0	–	–	–
	HM1022	29	4	13.79	12	5.50	2.50	10
	HM2211	18	0	0	–	–	–	–
	HM6711	17	0	0	–	–	–	–
HLP53	HM1404	19	0	0	–	–	–	–
	HM108	18	0	0	–	–	–	–
	HM1343	20	0	0	–	–	–	–
	HM1022	20	1	5.00	0	–	–	–
	HM2211	17	0	0	–	–	–	–
	HM6711	18	0	0	–	–	–	–
HLP72	HM1404	19	0	0	–	–	–	–
	HM108	16	0	0	–	–	–	–
	HM1343	21	0	0	–	–	–	–
	HM1022	18	0	0	–	–	–	–
	HM2211	16	0	0	–	–	–	–
	HM6711	20	0	0	–	–	–	–
*C. moschata*	*C. pepo*							
HM1404	HLP36	19	0	0	–	–	–	–
	HLP44	17	1	5.88	0	–	–	–
	HLP53	21	1	4.76	0	–	–	–
	HLP72	20	0	0	–	–	–	–
HM108	HLP36	18	0	0	–	–	–	–
	HLP44	18	0	0	–	–	–	–
	HLP53	20	1	5.00	0	–	–	–
	HLP72	17	1	5.88	0	–	–	–
HM1343	HLP36	20	1	5.00	0	–	–	–
	HLP44	21	2	9.52	0	–	–	–
	HLP53	23	8	34.78	8	0	–	–
	HLP72	20	1	5.00	0	–	–	–
HM1022	HLP36	19	0	0	–	–	–	–
	HLP44	20	0	0	–	–	–	–
	HLP53	17	0	0	–	–	–	–
	HLP72	18	0	0	–	–	–	–
HM2211	HLP36	21	1	4.76	0	–	–	–
	HLP44	18	0	0	–	–	–	–
	HLP53	16	0	0	–	–	–	–
	HLP72	19	0	0	–	–	–	–
HM6711	HLP36	18	1	5.55	0	–	–	–
	HLP44	19	1	5.26	0	–	–	–
	HLP53	19	0	0	–	–	–	–
	HLP72	20	4	20.00	6	0	–	–
Total		916	51	5.57				81

### Embryo rescue

**Figure 1:**
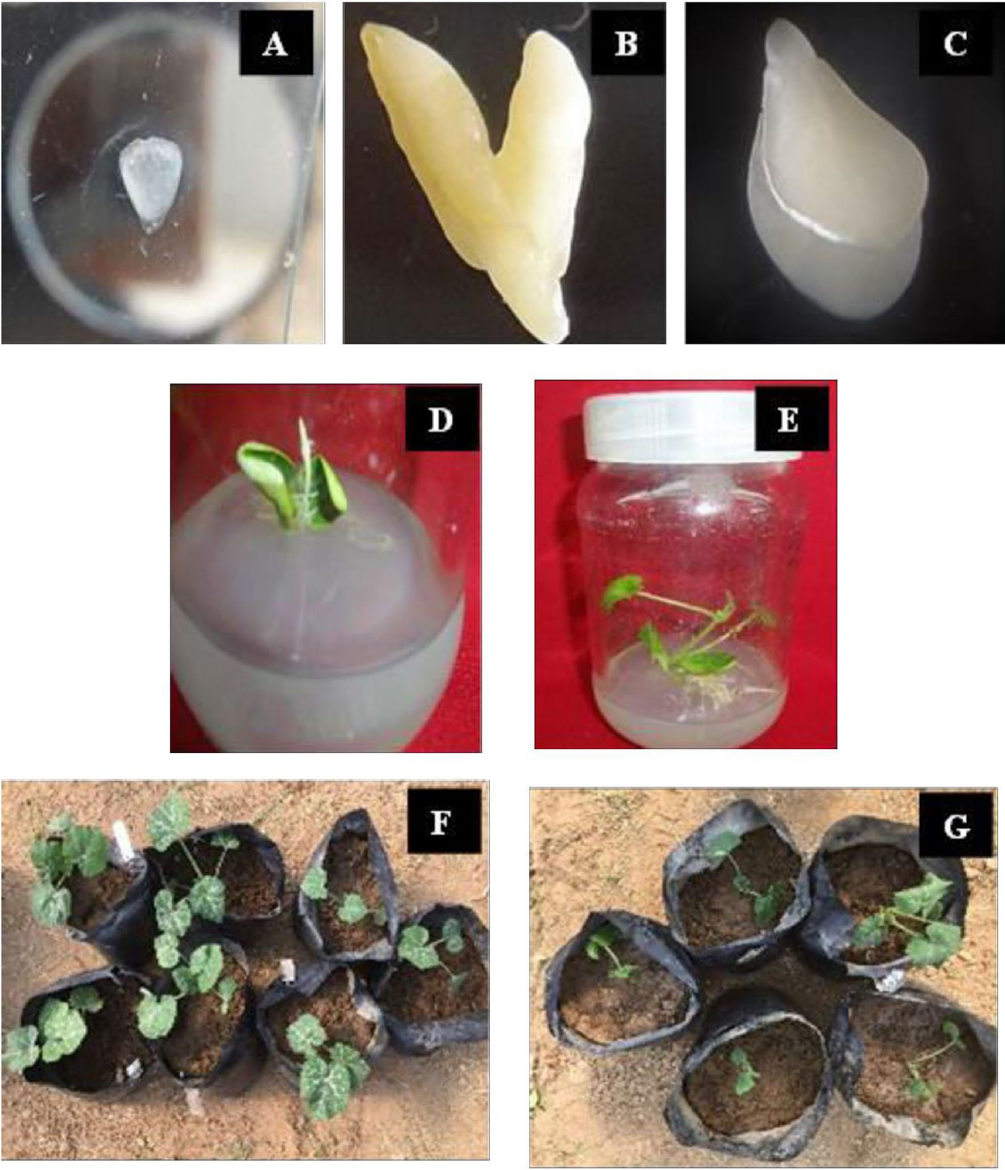
Identification of embryo developmental stages following interspecific hybridization and plantlet formation. (**A**) Embryo at heart stage, (**B**) Embryo at torpedo stage, (**C**) Embryo at cotyledonary stage, (**D**) Ovule germination, (**E**) Plantlet formation from cotyledonary staged embryos, (**F**,**G**) Putative interspecific hybrid plants acclimated in soil.

Keeping in view the above observations of incompatibility between *C. moschata* (as female parent) and *C. pepo* (as male parent), attempts were made for embryo rescue after different days of pollination (DAP). The heart shape embryos were identified after 11 to 13 DAP followed by detection of torpedo shape at 14 to 16 DAP and cotyledonary shape at 17 to 19 DAP (Fig. 1A–C). The cultured embryos did not respond up to 20 days, then turned brown and eventually decayed. Consequently, ovule culture was followed. The ovules isolated at 17 to 19 DAP displayed up to 36.00% germination (Table 2, Fig. 1D,E), whereas, the ovules cultured at 11 to 13 and 14 to 16 DAP had lower (2.00–6.00%) ovule germination (Table 2). Only the ovules cultured at 17 to 19 DAP displayed plantlet development up to 21.33% (Table 2). Total seven and five plants were acclimated from the cross of HM1343 × HLP53 and HM6711 × HLP72, respectively (Fig. 1F,G).

**Table 2:** Interspecific hybridization between *C. moschata* × *C. pepo* through ovule culture.

Cross combination	DAP	Ovule germination (%)	Plantlet development (%)	Number of plants acclimated	Success (%)
HM1343 × HLP53	11–13	3.33 (0.18)^c^	0.00 (0.00)^c^	–	–
	14–16	6.00 (0.24)^c^	0.00 (0.00)^c^	–	–
	17–19	36.00 (0.64)^a^	21.33 (0.48)^a^	7	4.67
HM6711 × HLP72	11–13	2.00 (0.11)^c^	0.00 (0.00)^c^	–	–
	14–16	4.00 (0.20)^c^	0.00 (0.00)^c^	–	–
	17–19	24.00 (0.51)^b^	11.33 (0.34)^b^	5	3.33
Total				12	

*DAP:* days after pollination.

Mean values with different letters in column are significantly different at *P* ≤ 0.05 according to Tukey's HSD (Honest Significant Difference) test.

Figures in parentheses are the means of arcsine transformed values.

### Molecular characterization of putative interspecific hybrids

**Figure 2:**
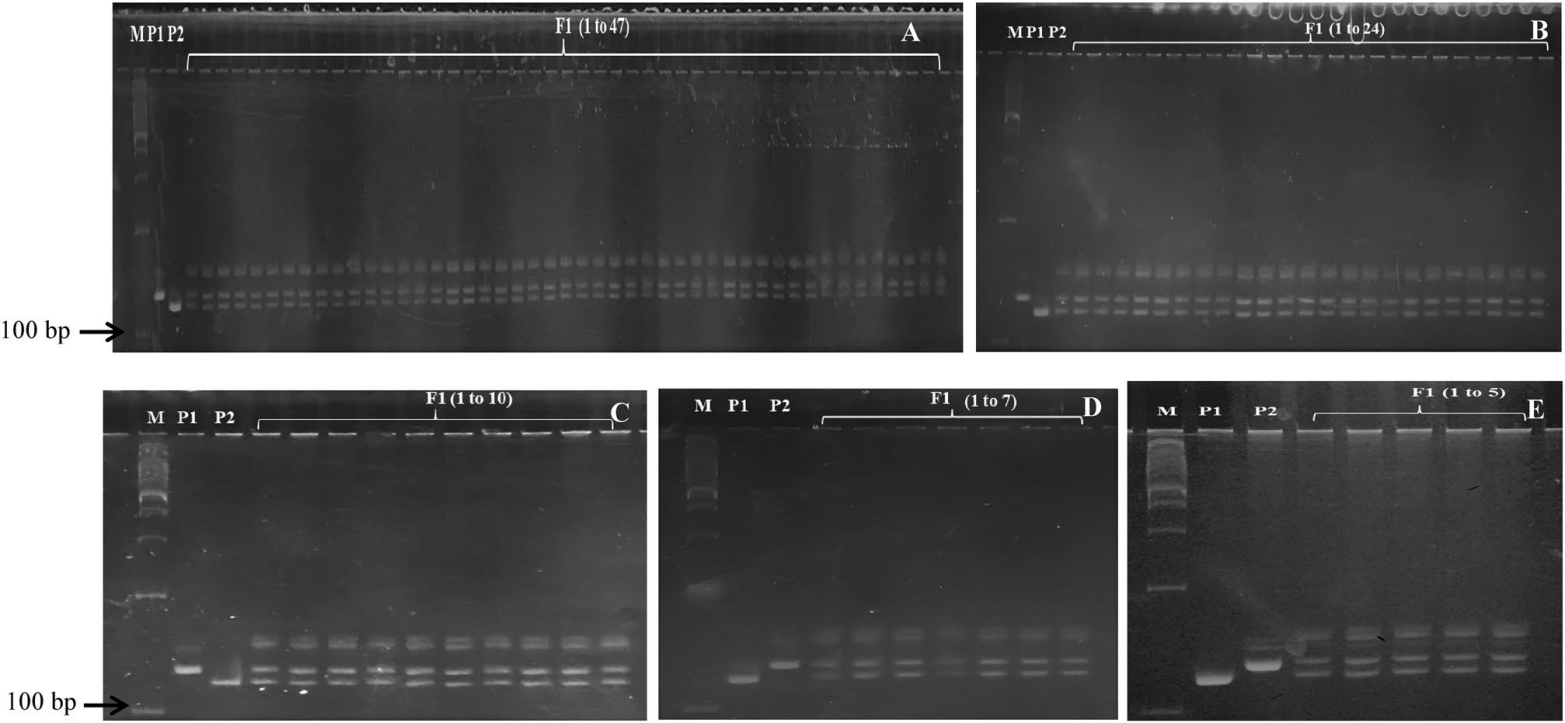
Molecular characterization of putative interspecific hybrids by SSR marker, CMTp182 with detection of 138 bp amplicon in crosses *C. pepo* × *C. moschata*: (**A**) HLP36 (P_1_) × HM1343 (P_2_), (**B**) HLP36(P_1_) × HM1022 (P_2_), (**C**) HLP44 (P_1_) × HM1022 (P_2_) and *C. moschata* × *C. pepo*: (**D**) HM1343 (P_1_) × HLP53 (P_2_), (**E**) HM6711(P_1_) × HLP72 (P_2_). M standard 100 bp DNA ladder.

Genomic DNA of putative interspecific hybrids developed by conventional pollination (HLP36 × HM1343, HLP36 × HM1022 and HLP44 × HM1022) and ovule culture (HM1343 × HLP53 and HM6711 × HLP72) along with their parents were examined for the assessment of hybridity using SSR markers. Polymorphism between *C. pepo* (hull-less) and *C. moschata* (hulled) parents was detected using SSR markers linked to hull-less seed trait^20^ and found CMTp182, CMTm47, and CMTm261 polymorphic between parents leading to amplification of 138, 143 and 228 bp fragments, respectively. From these, a marker viz., CMTp182 was used for the confirmation of hybridity of interspecific F_1_ plants and it amplified the heterozygous fragments of 138 bp corresponding to hull-less seed trait (Fig. 2).

### Morphological characterization

#### Qualitative traits

*C. pepo* and *C. moschata* used as parents in present study differed for qualitative traits, such as leaf blade margin, leaf blade silver patches, stem shape, fruit shape, fruit skin colour pattern and seed coat colour (Table 3). The leaf blade margin was moderately or strongly incised in *C. pepo* and entire or very weakly incised in *C. moschata*. In contrast, the interspecific hybrids possessed the weakly incised leaf blade margins, indicated the hybridity (Supplementary Fig. 1). *C. moschata* genotypes i.e. HM1343 and HM1022 displayed silver-grey patches in the axil of leaf veins and similar patches were observed in leaves of interspecific F_1_ hybrids, where these two were used as a parent in hybridization. The presence of silver-grey patches in axil of leaf veins indicated it to be dominant over the absence of silver-grey patches. The F_1_ hybrids of all interspecific cross-combinations displayed presence of stem, peduncle and leaf pubescence similar to both parents, however, the F_1_ hybrids were more inclined towards the maternal parent for stem shape, fruit shape, immature fruit skin colour, fruit skin lustre, fruit skin colour pattern and seed coat colour.

**Table 3:** Morphological (qualitative) characterization of interspecific hybrids and their parents.

Parentage	Stem shape	Stem pube-scence	Leaf blade margin	Leaf blade silver patches	Leaf pube-scence	Peduncle pube-scence	Fruit shape	Fruit shape at peduncle end	Fruit shape at blossom end	Immature fruit skin colour	Fruit skin lustre	Fruit skin colour pattern	Mature fruit skin colour	Seed coat colour
*C. pepo*														
HLP36	Angular	Present	Moderately or strongly incised	Absent	Present	Present	Spherical	Raised	Flat	Medium green	Matt	Uniform	Yellowish	Green
HLP44	Angular	Present	Moderately or strongly incised	Absent	Present	Present	Flat round	Moderately depressed	Depressed	Dark green	Glossy	Uniform	Yellowish	Green
HLP53	Angular	Present	Moderately or strongly incised	Absent	Present	Present	Spherical	Raised	Flat	Light green	Intermediate	Uniform	Yellowish	Green
HLP72	Angular	Present	Moderately or strongly incised	Absent	Present	Present	Spherical	Raised	Flat	Medium green	Glossy	Uniform	Yellowish	Green
Interspecific hybrids														
HLP36 × HM1343	Angular	Present	Weakly incised	Present	Present	Present	Spherical	Raised	Flat	Medium green	Matt	Uniform	Yellowish	Green
HLP36 × HM1022	Angular	Present	Weakly incised	Present	Present	Present	Spherical	Raised	Flat	Medium green	Matt	Uniform	Yellowish	Green
HLP44 × HM1022	Angular	Present	Weakly incised	Present	Present	Present	Flat round	Moderately depressed	Depressed	Dark green	Glossy	Uniform	Yellowish	Green
HM1343 × HLP53*	Round	Present	Weakly incised	Present	Present	Present	Flat round	Strongly depressed	Depressed	Dark green	Intermediate	Mottled	Yellowish	Cream
HM6711 × HLP72*	Angular	Present	Weakly incised	Absent	Present	Present	Flat round	Moderately depressed	Depressed	Dark green	Glossy	Mottled	Yellowish	Cream
*C. moschata*														
HM1343	Round	Present	Entire or very weakly incised	Present	Present	Present	Flat round	Strongly depressed	Depressed	Dark green	Intermediate	Mottled	Yellowish	Cream
HM1022	Round	Present	Entire or very weakly incised	Present	Present	Present	Flat round	Strongly depressed	Depressed	Dark green	Intermediate	Mottled	Yellowish	Cream
HM6711	Angular	Present	Entire or very weakly incised	Absent	Present	Present	Flat round	Moderately depressed	Depressed	Dark green	Glossy	Mottled	Yellowish	Cream

*Derived through ovule culture.

#### Quantitative traits

*C. pepo* and *C. moschata* were also divergent for number of quantitative traits, such as vine and internode length, number of primary branches, leaf blade length and width, petiole and peduncle length, days to flowering, node number of first female flower, pollen viability (%), number of ridges per fruit, fruit weight and diameter, flesh thickness and number of seeds formed per fruit (Table 4). Their interspecific hybrids were categorized from Family I to Family V. Each family comprised of interspecific hybrid along with male and female parent for better understanding of trait analysis. Family I to Family III indicating the interspecific hybrids from *C. pepo* × *C. moschata* were obtained by conventional pollination and Family IV to Family V of *C. moschata* × *C. pepo* by ovule culture. *C. pepo* possessed bush growth habit (51.27–65.33 cm) along with lower internodal length (4.50–5.67 cm) except HLP36. While, *C. moschata* which grows as a vine (186.67–317.93 cm) exhibited higher internodal length (7.57–15.17 cm), except line HM6711. The interspecific hybrids achieved intermediate vine length (58.89–271.07 cm) with appearance of bushy plants, where one of the parents was of bush growth habit like bush × vine (Family III), vine × bush (Family IV) and bush × bush (Family V). This indicated the dominance of bush growth habit over the vine and similar trend was noticed for internodal length (4.38–10.00 cm) of the main vine in interspecific hybrids. However, transgressive hybrids were recorded in Family V for attaining more number of primary branches (5.02), followed by other interspecific crosses (2.67–3.33). The leaf blade length (10.31–12.97 cm) and width (12.60–16.32 cm) of F_1_ hybrids were significantly enhanced from their respective female parent (7.36–11.80 cm, 9.40–16.32 cm) with exception of leaf blade length in Family I. In addition, interspecific F_1_ hybrids obtained from *C. pepo* × *C. moschata* revealed earliness for days to flowering (30.00–32.25) and number of nodes (6.28–7.78) of first female flower having statistically similarity with the maternal parent (26.07–26.38 days to flowering, 4.61–6.43 node number), whereas, in reciprocals these expressions were intermediate (31.87–32.65 days to flowering, 4.78–6.60 node number). The pollen viability of interspecific hybrids was observed significantly lower (66.70–77.33%) than the parents (87.60–91.26%). Fruiting traits, such as number of ridges (0.00–9.80), polar and equatorial fruit diameter (7.57–14.31 cm, 10.77–14.77 cm) and flesh thickness (1.90–2.90 cm) were more inclined towards maternal parents (0.00–11.08, 6.70–14.62 cm and 10.73–14.45 cm, 1.82–3.15 cm). The fruit weight of *C. pepo* × *C. moschata* hybrids (0.75–0.85 kg) was statistically similar to their female parent (0.65–0.72 kg), whereas, the reciprocal crosses revealed the intermediacy (0.61–0.71 kg). Numbers of seeds per fruit obtained from interspecific F_1_ plants were ranged from 25.33 to 89.67 compared with 94.67 to 316.67 of the parents. The interspecific F_1_ hybrids revealed divergence from their parents with respect to morphological traits, indicating the hybridity.

**Table 4:** Morphological (quantitative) characterization of interspecific hybrids and their parents.

Parentage	Vine length (cm)	Inter-nodal length (cm)	Number of primary branches	Leaf blade length (cm)	Leaf blade width (cm)	Petiole length (cm)	Peduncle length (cm)	Days to 50% flowering after transplanting	Node number at which first female flower appeared	Pollen viability (%)	No. of ridges per fruit	Polar diameter of fruit (cm)	Equatorial diameter of fruit (cm)	Flesh thickness (cm)	Fruit weight (kg)	No. of seeds per fruit
Family I																
HLP36	199.33^b^	11.50^a^	1.67 (1.28)^b^	11.44^a^	13.68^b^	9.57^c^	4.46^b^	26.07 (5.10)^b^	6.43 (2.53)^b^	89.01 (1.24)^a^	0.00	14.62^a^	12.46^b^	1.82^b^	0.65^b^	155.00 (12.43)^b^
HLP36 × HM1343	244.80^a^	9.17^ab^	3.33 (1.79)^a^	10.49^b^	15.01^a^	10.93^b^	4.45^b^	30.00 (5.47)^b^	6.72 (2.59)^b^	77.33 (1.07)^b^	0.00	14.26^ab^	12.53^b^	1.90^b^	0.75^b^	89.67 (9.42)^c^
HM1343	256.53^a^	7.57^b^	3.20 (1.82)^a^	9.35^c^	12.56^c^	14.36^a^	6.08^a^	39.07 (6.25)^a^	12.12 (3.48)^a^	90.03 (1.25)^a^	9.80	12.33^b^	15.17^a^	3.16^a^	1.17^a^	282.00 (16.78)^a^
Family II																
HLP36	199.33^f^	11.50^e^	1.67 (1.28)^e^	11.44^f^	13.68^f^	9.57^f^	4.46^e^	26.07 (5.10)^e^	6.43 (2.53)^e^	89.01 (1.24)^d^	0.00	14.62^e^	12.46^e^	1.82^e^	0.65^e^	155.00 (12.43)^e^
HLP36 × HM1022	271.07^e^	10.00^e^	2.67 (1.63)^d^	12.73^e^	16.19^e^	11.71^e^	5.56^e^	30.00 (5.47)^e^	7.78 (2.79)^e^	73.87 (1.03)^e^	0.00	14.31^de^	12.76^e^	2.05^e^	0.84^e^	63.00 (7.90)^f^
HM1022	317.93^d^	15.17^d^	3.07 (1.75)^d^	14.39^d^	17.35^d^	17.18^d^	12.54^d^	40.20 (6.34)^d^	13.92 (3.73)^d^	90.17 (1.26)^d^	11.08	16.37^d^	19.20^d^	3.17^d^	2.38^d^	316.67 (17.78)^d^
Family III																
HLP44	51.27^h^	4.50^i^	3.34 (1.83)^gh^	11.80^i^	14.51^i^	8.22^i^	5.57^h^	26.38 (5.13)^i^	4.61 (2.14)^h^	87.60 (1.21)^g^	9.00	11.24^h^	14.45^h^	2.59^h^	0.72^h^	134.33 (11.56)^h^
HLP44 × HM1022	68.87^h^	7.00^h^	3.71 (1.93)^g^	12.97^h^	16.32^h^	15.22^h^	5.66^h^	32.25 (5.67)^h^	6.28 (2.50)^h^	70.40 (1.00)^h^	9.80	11.32^h^	14.77^h^	2.62^h^	0.85^h^	49.00 (6.99)^i^
HM1022	317.93^g^	15.17^g^	3.07 (1.75)^h^	14.39^g^	17.35^g^	17.18^g^	12.54^g^	40.00 (6.34)^g^	13.92 (3.73)^g^	90.17 (1.26)^g^	11.08	16.37^g^	19.20^g^	3.17^g^	2.38^g^	316.67 (17.78)^g^
Family IV																
HM1343	186.67^j^	7.71^j^	3.20 (1.79)^j^	9.26^l^	12.47^l^	14.01^j^	6.01^j^	40.47 (6.36)^j^	10.30 (3.21) ^j^	90.03 (1.25) ^j^	9.80	10.31^jk^	12.64^j^	3.15^j^	1.00^j^	145.33 (12.03)^j^
HM1343 × HLP53*	109.67^k^	6.17^jk^	3.03 (1.74)^j^	10.31^k^	13.74^k^	12.22^j^	5.08^j^	31.87 (5.64)^jk^	6.60 (2.57) ^k^	66.70 (0.95) ^k^	9.50	10.06^k^	11.08^j^	2.90^jk^	0.71^k^	25.33 (5.02)^k^
HLP53	60.33^l^	5.33^k^	3.12 (1.76)^j^	11.37^j^	14.84^j^	12.15^j^	4.56^j^	26.27 (5.12)^k^	4.05 (2.01) ^l^	89.92 (1.25) ^j^	0.00	11.53^j^	12.04^j^	2.42^k^	0.75^k^	99.33 (9.92)^j^
Family V																
HM6711	36.04^n^	3.00^o^	2.02 (1.41)^o^	7.36^n^	9.40^n^	6.34^n^	1.65^n^	37.35 (6.11)^m^	2.42 (1.55)^n^	91.26 (1.27)^m^	9.32	6.70^n^	10.73^n^	2.51^m^	0.56^n^	94.67 (9.69)^n^
HM6711 × HLP72*	58.89^m^	4.38^n^	5.02 (2.24)^m^	11.31^m^	12.60^m^	9.16^mn^	2.26^m^	32.65 (5.71)^mn^	4.78 (2.18)^m^	70.55 (1.00)^n^	9.22	7.57^n^	10.77^n^	2.63^m^	0.61^mn^	32.00 (5.65)^o^
HLP72	65.33^m^	5.67^m^	3.03 (1.74)^n^	12.20^m^	14.50^m^	12.40^m^	2.58^m^	27.68 (5.26)^n^	5.47 (2.34)^m^	89.37 (1.24)^m^	0.00	13.76^m^	12.38^m^	2.14^m^	0.73^m^	145.33 (12.03)^m^

Mean values with different letters in a respective family number's column are significantly different at *P* ≤ 0.05 according to Tukey's HSD (Honest Significant Difference) test.

Family numbers are designated with different letters as: Family I: a–c, Family II: d–f, Family III: g–i, Family IV: j–l, Family V: m–o.

Figures in parentheses are the means of square root and arc sine transformed values.

*Derived through ovule culture.

### Micromorphological characterization

The micromorphological traits, such as trichomes and stomatal aperture on abaxial leaf surface of parents and their interspecific F_1_ plants were examined through scanning electron micrography (Table 5, Supplementary Figs. 2 and 3). The trichome density of *C. pepo* varied from 3.00 to 3.40 per 100 μm^2^, *C. moschata* from 3.60 to 4.00 per 100 μm^2^, and their interspecific F_1_ hybrids were significantly enhanced from their respective female parent in Family II (4.40 per 100 μm^2^), transgressive to both the parents in Family III (4.80 per 100 μm^2^) and intermediate to both the parents in Family I, IV and V (3.60, 3.40 and 3.40 per 100 μm^2^), indicated hybridity. Further, the hybridity was assessed with trichome length, which revealed that *C. moschata* exhibited longer trichomes (286.89–485.73 µm) in comparison to *C. pepo* (178.78–364.66 µm). While their interspecific F_1_ hybrids were intermediate in Family II (310.01 µm) and III (434.25 µm), transgressive in Family IV (404.62 µm), and statistically different from female parent in Family I (284.13 µm) and from male parent in Family V (305.48 µm) for trichome length. Trichome width and stomatal complex length were also analyzed for the confirmation of hybridity, however results were non-significant between parents and their interspecific hybrids except Family I and II for trichome width.

**Table 5:** Micromorphological characterization of interspecific hybrids and their parents.

Parentage	Trichome density (per 100 µm^2^)	Trichome length (µm)	Trichome width (µm)	Stomatal complex length (µm)
Family I				
HLP36	3.20^b^	178.78^b^	27.27^b^	6.87^a^
HLP36 × HM1343	3.60^ab^	284.13^a^	28.16^b^	6.24^a^
HM1343	3.90^a^	318.65^a^	35.64^a^	5.16^a^
Family II				
HLP36	3.20^e^	178.78^f^	27.27^e^	6.87^d^
HLP36 × HM1022	4.40^d^	310.01^e^	39.53^d^	7.35^d^
HM1022	4.00^d^	485.73^d^	38.85^d^	8.36^d^
Family III				
HLP44	3.40^i^	310.56^i^	39.54^g^	7.90^g^
HLP44 × HM1022	4.80^g^	434.25^h^	38.15^g^	8.04^g^
HM1022	4.00^h^	485.73^g^	38.85^g^	8.36^g^
Family IV				
HM1343	3.90^j^	318.65^k^	35.64^j^	5.16^j^
HM1343 × HLP53*	3.40^jk^	404.62^j^	39.14^j^	5.72^j^
HLP53	3.00^k^	284.28^k^	35.79^j^	5.52^j^
Family V				
HM6711	3.60^m^	286.89^n^	37.63^m^	5.65^m^
HM6711 × HLP72*	3.40^mn^	305.48^n^	36.45^m^	6.20^m^
HLP72	3.00^n^	364.66^m^	36.99^m^	4.67^m^

*Derived through ovule culture.

Mean values with different letters in a respective family number's column are significantly different at *P* ≤ 0.05 according to Tukey's HSD (Honest Significant Difference) test.

Family numbers are designated with different letters as: Family I: a–c, Family II: d–f, Family III: g–i, Family IV: j–l, Family V: m–o.

### Development and characterization of F_2_ populations for hull-less seed trait

All the F_1_ seeds obtained from the successful interspecific crosses between *C. pepo* × *C. moschata* were sown and then selfed. Approximately 80% of the interspecific F_1_ plants set the selfed fruits and each fruit had 40–50 seeds. The F_2_ populations of family I, family II and family III were raised from half of the fruits because pumpkin is a wider spaced crop (3 m × 60 cm) which is the major constraint in developing large population. However, all the seeds of each selected fruit were grown so that segregation ratios do not get affected. For the interspecific F_1_ hybrids of family IV and family V developed through ovule culture, all the F_2_ seeds were grown, selfed and then phenotyped for seed type.

**Figure 3:**
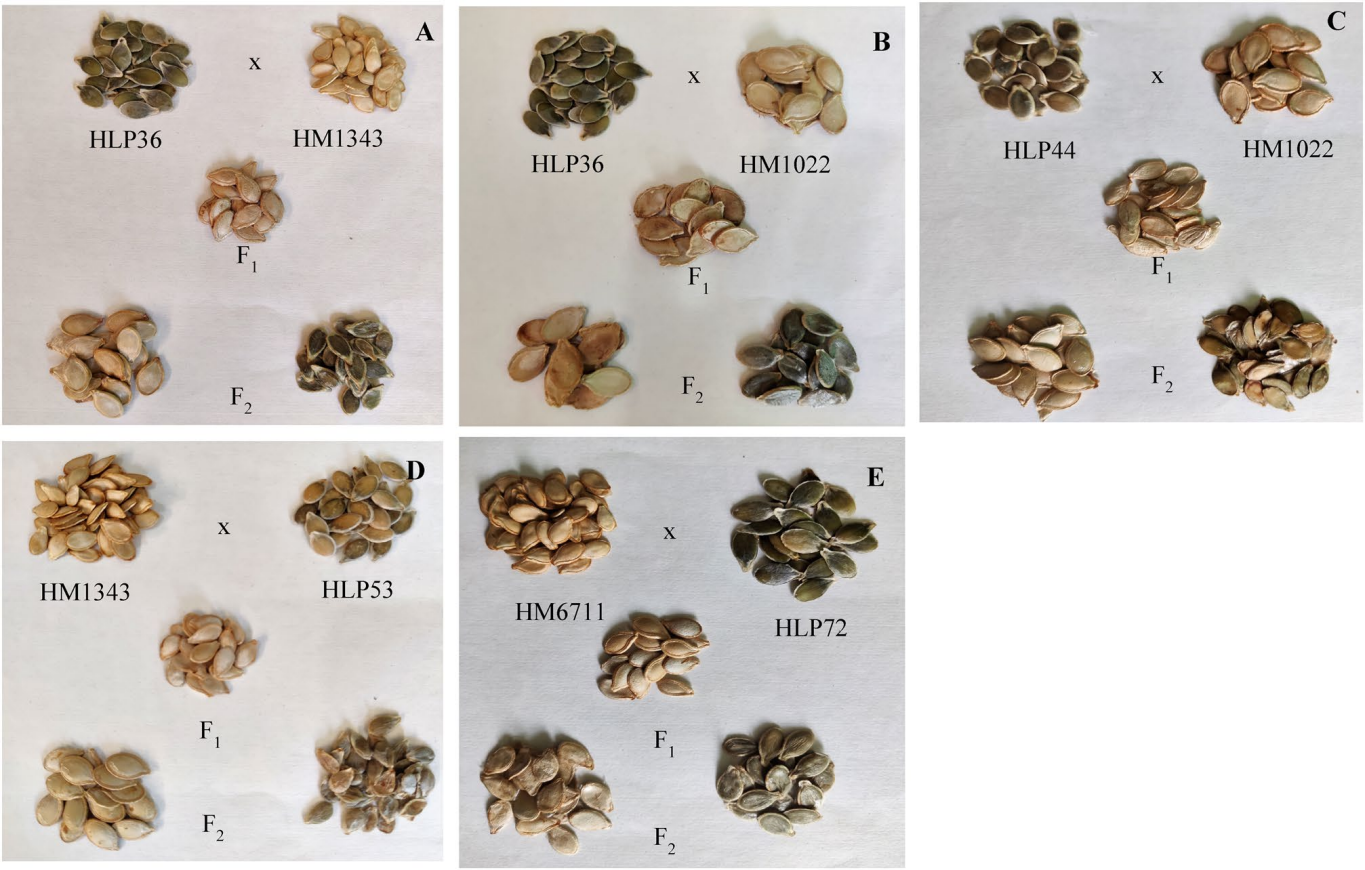
Representing phenotype of seeds used in the study. (**A**) Family I (HLP36 x HM1343), (**B**) Family II (HLP36 x HM1022), (**C**): Family III (HLP44 x HM1022), (**D**) Family IV (HM1343 x HLP53), (**E**) Family V (HM6711 x HLP72); F_1_: Interspecific F_1_ hybrid of respective cross; F_2_: Hulled and hull-less representatives of F_2_ generation.

The seeds collected from interspecific F_2_ plants from all cross-combinations (Family I to Family V) were phenotyped (Table 6, Fig. 3). The hulled nature of seeds in F_1_ generation indicated recessive nature of hull-less trait. The F_2_ populations of HLP36 × HM1343 (Family I), HLP36 × HM1022 (Family II), HLP44 × HM1022 (Family III) and HM6711 × HLP72 (Family V) were segregated into 189, 53, 21, 36 and 26 hulled and 51, 10, 2, 4 and 5 hull-less seeded plants, respectively. Chi-square analysis indicated a mendelian ratio of 3:1 (hulled:hull-less) as best fit in this hypothesis, which suggested that hull-less trait is governed by single gene. However, F_2_ population of HM1343 × HLP53 (Family IV) showed the segregation distortion w.r.t ratio 3:1. Further, a representative of interspecific F_2_ population (72 plants) derived from HLP36 × HM1343 cross was genotyped for hull-less seed trait using SSR marker, CMTp182 to assess the marker-trait association. However, only 58 (80.55%) plants displayed the accurate seed phenotype as predicted by the genotype (Supplementary Fig. 4a,b).

**Table 6:** Segregation of hull-less seeds in F_1_ and F_2_ populations of interspecific crosses.

Interspecific cross-combinations	Phenotype of seeds from F_1_ plants	Total number of F_2_ seeds sown	Total number of F_2_ seedlings transplanted	Number of fruit set on F_2_ plants upon selfing	Chi-square (χ^2^) analysis		Tested ratio	χ^2^ cal	*P* value
					Phenotype of seeds from F_2_ plants				
					Hulled seeded plants	Hull-less seeded plants			
HLP36 × HM1343 (Family I)	Hulled	700	413	240	189	51	3:1	1.80	0.18^ ns^
HLP36 × HM1022 (Family II)	Hulled	308	197	63	53	10	3:1	2.80	0.09^ ns^
HLP44 × HM1022 (Family III)	Hulled	161	102	23	21	2	3:1	3.26	0.07^ ns^
HM1343 × HLP53* (Family IV)	Hulled	170	91	40	36	4	3:1	4.80	0.03
HM6711 × HLP72* (Family V)	Hulled	150	85	31	26	5	3:1	1.30	0.25^ ns^

*Derived through ovule culture.

At 5% level of significance and 1 df, χ^2^ = 3.84.

*ns* non-significant.

## Discussion

Hull-less seed trait is popular in nut and bakery industries for easy access to nutritionally rich oil and directly used as snacks. In tropics, this trait is available only in *C. pepo* background, which is sensitive to high temperature and mosaic virus, limiting its cultivation to spring season^5^. Contrarily *C. moschata* possesses all desirable attributes viz*.,* high yield, large seed cavity, wider adaptability to seasons and locations, resistance to mosaic virus but lacks hull-less seed trait^4,21^. In this study, hull-less seed trait was transferred from *C. pepo* to *C. moschata* through interspecific hybridization by overcoming interspecific incompatibility barrier using conventional pollination as well as ovule culture approach.

Conventional pollination was successful, when *C. pepo* was used as a female parent, but with genotype specificity. Differences in pollen tube growth and style length of genotypes described variable fertilization observed between *C. moschata* and *C. pepo* crosses^9^.

The interspecific hybrids were assessed for hybridity through molecular, morphological and micromorphological markers. For molecular confirmation of interspecific hybridity, eight SSR markers (CMTm239, CMTm115, CMTp151, CMTp58, CMTm261, CMTp182, CMTm47 and CMTp39) linked to the hull-less locus (*h*) on Linkage Group 9 of *C. pepo*^20^ were used. However, only three viz., CMTp182, CMTm261 and CMTm47 were polymorphic and linked to hull-less trait (genetic distance > 25 cM, unpublished data) in the *C. pepo* populations available at PAU, Ludhiana. Among them a marker viz., CMTp182 was found closest to the hull-less locus and had more per cent accuracy of genotypic data with phenotype. Therefore, we selected CMTp182 for the confirmation of hybridity of putative interspecific F_1_ plants. In support to this, our recently published manuscript^22^ identified a candidate genomic region linked to hull-less seed trait ranging from 1.80 to 3.86 Mb on chromosome 12 of *C. pepo* genome and also reported a linked KASP marker. Similar results are also reported by two other recently published studies in *C. pepo*^23,24^. Further, BLASTn results of polymorphic SSR markers with *C. pepo* (Zucchini) genome revealed that only CMTp182 had the significant hits on chromosome 12 in between the identified candidate genomic region (CMTp182 Forward: 2220598–2220615 and CMTp182 Reverse: 2220732–2220713). All of these results validated the use of CMTp182 for confirmation of interspecific hybridity among the crosses of hull-less *C. pepo* and hulled *C. moschata* parents.

The plantlets inheriting SSR alleles from both the parents, confirmed the hybridity. Similarly, the detection of heterozygous SSR alleles in F_1_ interspecific hybrids have been used as an indicator of hybridity in crosses of cultivated eggplant with its wild relatives for resistance to bacterial wilt, root-knot nematode and verticillium wilt^25,26^. Likewise, amplification of male and female parent specific alleles using SSR markers in F_1_ interspecific hybrids of *Brassica carinata* having black rot resistance with *B. oleracea,* was used to verify hybridity^27^.

The divergence in morphological traits of interspecific hybrids from their parents is another indicator suggesting the hybridity. The variations in morphological traits associated with productivity in F_1_ interspecific hybrids of *C. maxima* × *C. moschata* and prolonged photoperiods in *Cucumis sativus* × *C. hytivus* from their parents, was taken as an evidence of hybridization^12,28^. Similar observations were also recorded in interspecific hybrids of *Solanum*^29^ and *Brassica*^30^.

The divergent expression of interspecific hybrids from their parents for micromorphological leaf traits (trichomes and stomata) also substantiated hybridity. These leaf traits have been used for prediction of hybridity in number of species such as *Cupressus*^31^, *Mentha*^32^, *Pinus*^33^, *Quercus*^34^ and *Trifolium*^35^. High density trichomes might be associated with biotic and abiotic stress resistance due to antimicrobial secondary metabolites^36,37^. The association of high density trichomes with plant resistance has been demonstrated in pumpkin for powdery mildew^38^ and high temperature tolerance^39^.

Molecular, morphological and micromorphological characterization confirmed the development of interspecific hybrids in present study. Phenotypically all F_1_s had hulled seeds. Thus, to unveil the transfer of hull-less seed trait, subsequent segregating generations (F_2_) were developed and subjected to goodness of fit test. Genetic ratios revealed that the hull-less seed trait (*h*) is governed by a single recessive gene except in interspecific cross, HM1343 × HLP53 (Family IV). These results are similar to the previous reports in *C. pepo* by Stuart^40^, Zraidi et al.^41^ and Lelley et al.^3^. More recently, along with monogenic recessive inheritance, hull-less seed trait has been mapped to chromosome 12 of *C. pepo* genome with ‘NST1’ (*Cp4.1Lg12g04350*) as the putative candidate gene^22–24^. However, segregation distortion observed in Family IV, might be due to incomplete representation of whole F_2_ population in chi square analysis. This could be the consequence of differential fertilization response of each F_2_ plant due to pollen-pistil incompatibilities^42^. It has also been reported in several interspecific crosses of rice^43^, wheat^44^, cotton^45^ and potato^46^. The distortions observed in the F_2_ generation among marker-phenotype associations might be due to the physical distance of marker from hull-less responsible gene^22–24^. Moreover, these distortions are of regular occurrence in early generations of wide crosses due to genomic instabilities or meiotic distortions^47,48^. Similar discrepancies for phenotype prediction were observed with genotypic data on introgressing black rot resistance in *Brassica*^27^.

## Conclusion

The interspecific hybrids for transfer of hull-less seed trait from *C. pepo* to *C. moschata* were developed and characterized through molecular markers, morphological and micromorphological traits. The presence of hull-less seeds among F_2_ populations substantiated the successful transfer of hull-less trait to *C. moschata.* The availability of this trait in high yielding, virus resistant and wider adaptable line(s) of *C. moschata* would enhance the productivity of hull-less seeds, and will make possible to grow the crop round the year in tropical and subtropical regions.

## Material and methods

### Plant material

Interspecific crosses between *C. pepo* and *C. moschata* were attempted under natural conditions at Vegetable Research Farm, Department of Vegetable Science, Punjab Agricultural University (PAU), Ludhiana (30° 54′ N and 70° 45′ E) during spring 2017. Genotypes used in the study possessing diverse phenotypic characters with their respective pedigree are given in Table 7. Thirty seeds per genotype were sown in pro-trays (98-hole capacity, 53.4 × 27.94 × 2.7 cm) using coco-peat based media in last week of January to second week of February, 2017. These pro-trays were placed in full sunlight, watered regularly and sprayed twice with N:P:K (19:19:19) for better seedling growth. Sowing dates were staggered three times at weekly interval for extending the availability of flowers. Three to four week old healthy seedlings at two true leaf stage were transplanted in the open field in March, 2017. It is stated that the plant material used in the present study complies with the Institutional guidelines. The hull-less seeded variety 'Lady Godiva' which was introduced from USA, having accession number EC 664187, dated 18/11/2009.

**Table 7:** *C. pepo* and *C. moschata* genotypes.

S. no.	Genotypes	Pedigree of genotypes	Characteristics		
			Seed coat type	Growth habit	Others
*C. pepo*					
1	HLP36*	WT-2012-36-231	Hull-less	Medium vine	Sparse vegetative growth, smaller fruit size and seed cavity and more sensitive to high temperature
2	HLP44*	WTP-2010-44-706	Hull-less	Bush	Medium sized dark green fruit
3	HLP53*	WT-4453-7	Hull-less	Bush	Medium sized light green fruit
4	HLP72*	WT-119-72	Hull-less	Bush	Prolific yielder
*C. moschata*					
1	HM1404**	PS-1404-2-1	Hulled	Long vine	Medium sized fruit and prolific yielder
2	HM108**	P-108-1213-1-1-1	Hulled	Long vine	Large sized fruit and seed cavity
3	HM1343**	P-1343-17-6-5-1	Hulled	Long vine	Medium sized fruit and tolerant to Yellow Vein Mosaic Virus
4	HM1022**	B-1022-2-2-5	Hulled	Long vine	Large sized fruit and seed cavity
5	HM2211**	CFR-2211-2	Hulled	Medium vine	Medium sized fruit
6	HM6711**	MVSR-6711-14-6-11	Hulled	Bush	Smaller seed cavity

*HLP: Hull-less *C. pepo*, **HM: Hulled *C. moschata.*

### Interspecific hybridization and embryo rescue

Interspecific crosses were attempted between *C. pepo* and *C. moschata* in reciprocal manner from end March to mid May, 2017 using controlled hand pollination. Fully developed unopened flower buds expected to open next morning were selected and covered with butter paper bags in the evening hours. In next morning between 6:00 and 8:00 a.m., freshly opened female flowers of one species were pollinated with pollen grains collected from male flowers of another species and vice-versa. The pollinated flowers were labeled and again covered with butter paper bags. This process was continued till the termination of flowering season. Fruits were harvested 45–50 days after hybridization to extract, wash, clean and dry seed for the storage.

The interspecific crosses between *C. moschata* × *C. pepo* i.e. HM1343 × HLP53 and HM6711 × HLP72 were again performed from March to May, 2018 followed by embryo rescue and ovule culture approach. The developing ovaries were collected daily after 10 to 20 days of pollination, brought to the Tissue Culture Laboratory, Department of Fruit Science, PAU. Initially, the collected ovaries were washed with running tap water and surface sterilized with 70% ethanol for 30 s followed by 20% (v/v) Clorox bleach along with 2 to 3 drops of Tween-20 for 30 min and washed thrice with sterile distilled water^17^. Subsequently, ovaries were dissected in a sterile petridish using sterilized forceps and scalpel blade inside a laminar airflow cabinet (Klenzoids, India). After that ovules were extracted from ovaries and eventually embryos were excised with sterilized sharp needle and scalpel under a stereozoom microscope. The distinct embryo developmental stages were observed. The excised embryos and ovules (50 each per replication) from both the cross-combinations were cultured on MS media^49^ supplemented with 0.01 IAA mg l^−1^ and 0.1 Kinetin mg l^−1^ along with 3% (w/v) sucrose and 0.8% (w/v) agar in jars (400 ml, Borosil) over three replicates at different days of pollination^18^. The media constituents used in the present study were acquired from HiMedia, India. The cultures were incubated at 25 ± 1 °C subjected to 16:8 h light:dark conditions. After 21–25 days, germinated ovules were subjected to sub-culturing on the same medium. Finally, the roots of plantlets taken out from the culture tubes were washed in tap water, dried on moist cotton and hardened by incubating at 20–25 °C under 16 h light for a week. These plantlets were transferred to polythene bags containing soil and farmyard manure in 1:1 ratio under poly-net house conditions during September 2018.

### Molecular characterization

The genomic DNA from tender leaf tissues collected from parents and their interspecific hybrids was isolated using cetyltrimethylammonium bromide method^50^. The isolated DNA was quantified using spectrophotometer and 0.8% agarose gel with standard uncut lambda DNA marker. The characterization for hybridity was carried out through eight SSR markers linked to hull-less seed trait (Table 8)^20^. Among the three polymorphic markers detected between *C. pepo* and *C. moschata,* SSR marker CMTp182, was selected for the confirmation of hybridity of interspecific F_1_ plants. The PCR mixture of total volume of 10 μl reaction comprised 3.5 μl of 2 × EmeraldAmp^®^ GT PCR Master Mix (Takara), 0.6 μl of 20 μM each forward and reverse primer, 2 μl template DNA (50 ng/μl) and sterile water. The mixtures were placed in thermocycler (Eppendorf, Germany) programmed with initial denaturation at 95 °C for 5 min, followed by 35 cycles of 4 min denaturation at 94 °C, primer annealing at 45–55 °C for 45 s and 1 min extension at 72 °C and the final extension at 72 °C was held for 5 min. The amplified products were resolved by electrophoresis in 6% (w/v) polyacrylamide gel at constant voltage 300 V/cm for 1–1.5 h and visualized under gel documentation system.

**Table 8:** SSR markers utilized for characterization of putative interspecific hybrids.

S. no.	Marker	Forward sequence (5′–3′)	Reverse sequence (5′–3′)	Expect size (bp)
1	CMTp182*	CACGAAGATTTGATGGCCTTA	GGATTGGGATGGTGAAGATG	138
2	CMTp39	GGCGAAAAGGAAGAACGAAT	TTTTTCTCCCCCTTCCACAT	132
3	CMTp58	TCGGAGAAACTCGACACTCC	TCCCAGCACCATCAGGATAC	102
4	CMTp151	CGGAGAAACTCGACACTCC	CCCAGCACCATCAGGATAC	99
5	CMTm115	AAGTCCACAACATGCAAACG	TCTCTTAATTGTTTCTCCCGATCT	99
6	CMTm239	CAAAGATCTGTTGTGTCAGAGT	GGAGAGTGGAGGAGGTAGAT	167
7	CMTm47*	TCCATTCCCAGATTTGAACG	CAGAGCCCACCTAACAGCAG	143
8	CMTm261*	GGTGGCCTCTGAACAATTTC	ACCTAACCAATGGGCATGAG	228

*Polymorphic markers.

### Morphological characterization

The parental genotypes and their interspecific hybrids were characterized for 29 morphological traits comprising 14 qualitative traits i.e. stem shape, stem pubescence, leaf blade margin, leaf blade silver patches, leaf pubescence, peduncle pubescence, fruit shape, fruit shape at peduncle end, fruit shape at blossom end, immature fruit skin colour, fruit skin lustre, fruit skin colour pattern, mature fruit skin colour, seed coat colour and 15 quantitative traits like vine length (cm), internodal length (cm), number of primary branches, leaf blade length (cm), leaf blade width (cm), petiole length (cm), peduncle length (cm), days to 50% flowering, node number at which first female flower appeared, number of ridges per fruit, polar diameter of fruit (cm), equatorial diameter of fruit (cm), flesh thickness (cm), fruit weight (kg) and number of seeds per fruit. These parameters were measured according to the NBPGR (National Bureau of Plant Genetic Resources) guidelines and PPV&FRA (Protection of Plant Varieties and Farmer's Right Act) descriptor^51^ (http://www.plantauthority.gov.in). Pollen viability (%) of parents and their interspecific hybrids was calculated at 50% flowering stage by dusting the mature pollen grains in 1% acetocarmine solution (HiMedia) and counting the number of round, stained pollen grains vs. shrivelled pollen grains in five microscopic fields under compound light microscope (Leica Bright Field microscope). The in-vitro generated plantlets of interspecific hybrids were compared with their respective parents under poly-net house conditions.

### Micromorphological characterization

The interspecific hybrids and their parents were scrutinized for micromorphological traits such as trichome density (per 100 µm^2^), trichome length and width (µm) and stomatal complex length (µm) on abaxial leaf surface using SEM (Scanning Electron Microscope) at Electron Microscopy and Nanoscience Laboratory, Department of Soil Science, PAU, Ludhiana. The third or fourth fully expanded leaves from tip per parent genotype and interspecific hybrids were taken for micrography. Initially, leaf surface was washed thoroughly with sterilized double distilled water to remove the adhering dust particles, followed by removal of excess water present on leaves using tissue paper. The square shaped leaf segments of approximately 10 mm^2^ size per leaf were excised from interveinal region of maximum leaf blade width. Further, processing steps involved the primary fixation of these leaf segments in 2.5% glutaraldehyde at 4 °C for 24 h, followed by draining of this fixative with three washings of 0.1 M sodium cacodylate buffer (pH 7.2–7.4) for 15 min at 4 °C. Then, leaf specimens were post-fixed in the 1% osmium tetraoxide (OsO_4_), followed by aforementioned washings of tissue samples with sodium cacodylate buffer to remove excess OsO_4_. The fixed tissues were further treated with graded ascending ethanol series (from 30 to 100%) for 15–20 min at room temperature and afterwards placed in vacuum dessicator. The dried samples were mounted on aluminium stubs using double sided carbon adhesive tape and then coated with 10–20 nm gold layer in an ion sputter coater (Hitachi E-1010). The micrographs were obtained using SEM (Hitachi S-3400 N, Japan) at 10–15 kV in back scattered electron imaging mode at different magnifications. The leaf traits were examined from standard area of 0.25 mm^2^ per leaf section with two to three randomly selected microscopic fields and data was measured using ImageJ.

### Development and characterization of F_2_ populations for hull-less seed trait

The interspecific F_1_ hybrids were selfed to generate F_2_ populations and both (F_1_ and F_2_) were phenotyped for hull-less seed trait. Healthy female and male flower of each respective interspecific F_1_ plant was covered with butter paper bag in the evening one-day prior to anthesis followed by pollination of former with latter in next morning (6:00–8:00 a.m.). These pollinated flowers were again covered with butter paper bags and tagged with jewel tags. Fruits were harvested after 45–50 days of pollination and seeds were extracted from these fruits, dried, and then phenotyped for hull-less seed trait. In the following season, these seeds were sown and selfing was performed. The seeds collected from F_2_ plants were phenotyped for hull-less seed trait. The phenotyping was categorized as: hulled seeds and hull-less seeds, according to Zraidi et al.^41^. Also, the interspecific F_2_ plants were genotyped for hull-less seed trait using SSR marker, CMTp182 to confirm the marker-trait association.

### Statistical analysis

The data pertaining to development of interspecific hybrids through ovule culture, morphological (quantitative traits) and micromorphological characterization of interspecific hybrids was statistically analyzed according to Tukey's HSD (Honest significant difference) test at *P* ≤ 0.05 using SPSS version 26.0 (IBM, Corp., Armonk, NY, USA). The data was normalized using arc sine and square root transformations. Chi-square test was used to evaluate the goodness of fit for hull-less seed trait in F_1_ and F_2_ generations of all interspecific crosses.

## Supplementary Information

Supplementary Figures.

## Data Availability

The datasets used and/or analysed during the current study are available from the corresponding author on reasonable request.
