# Supplementary material for: Interspecific hybridization for transfer of hull-less seed trait from Cucurbita pepo to C. moschata
Source: Sci Rep. 2023 Mar 21;13:4627. doi: 10.1038/s41598-023-29935-9 (PMC10030865; doi:10.1038/s41598-023-29935-9)
Supplement: Supplementary file 1 — Supplementary Figures. [file 41598_2023_29935_MOESM1_ESM.docx]

| 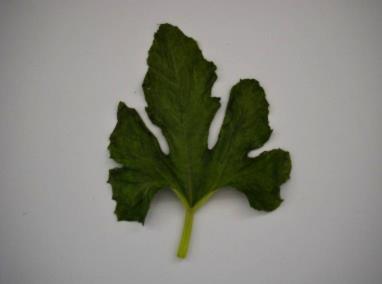  **A** | | **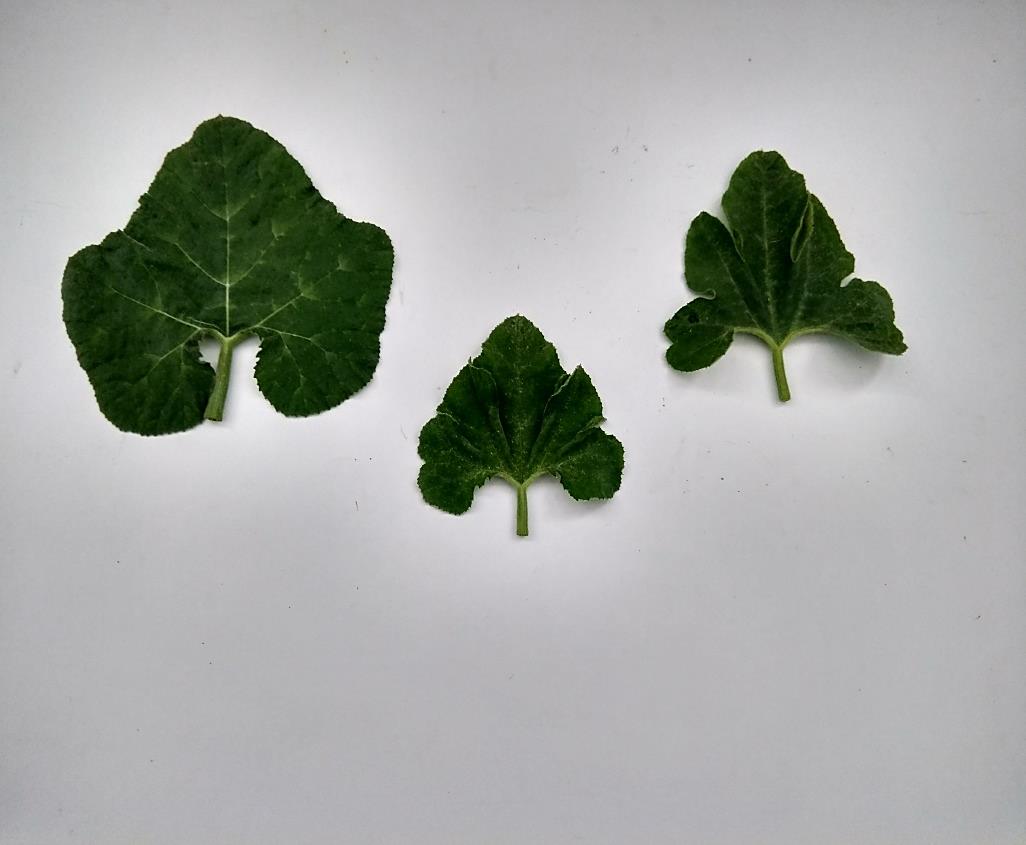**  **B** | | | **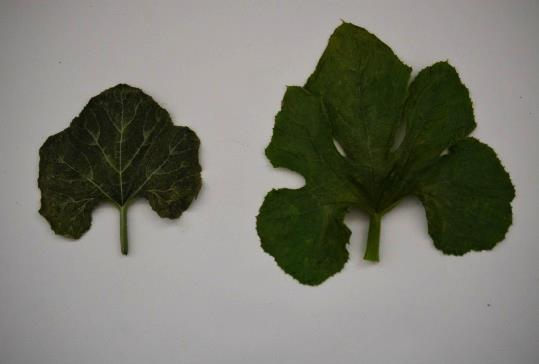**  **C** | | | **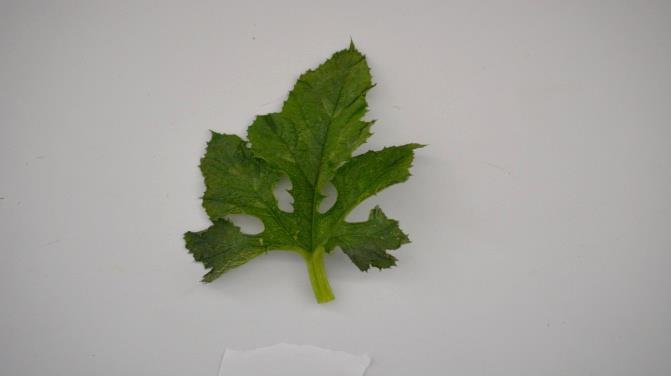**  **D** | |
| --- | --- | --- | --- | --- | --- | --- | --- | --- | --- |
| **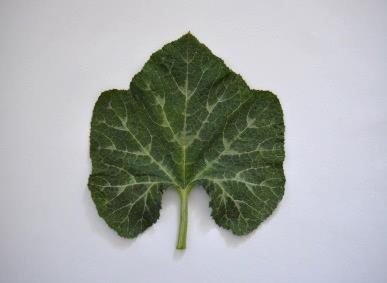**  **E** | | | **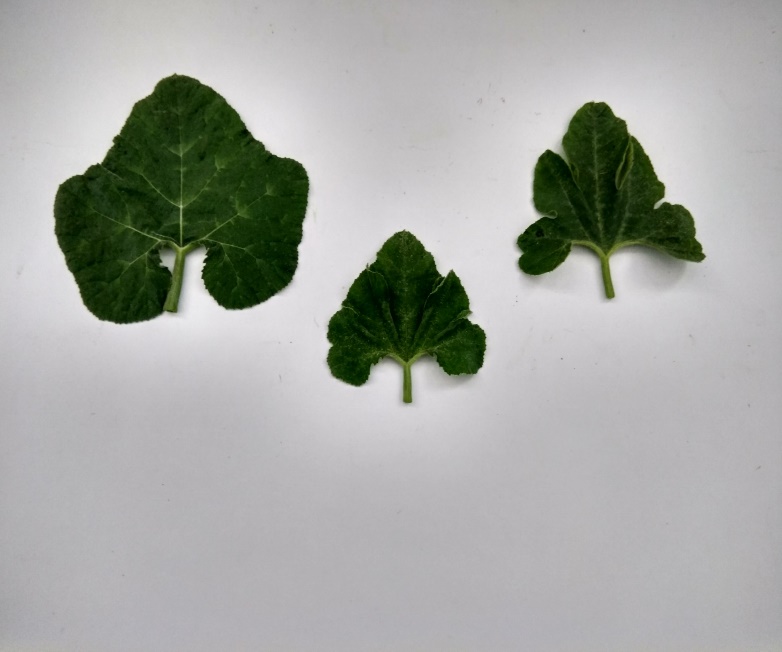**  **F** | | | | **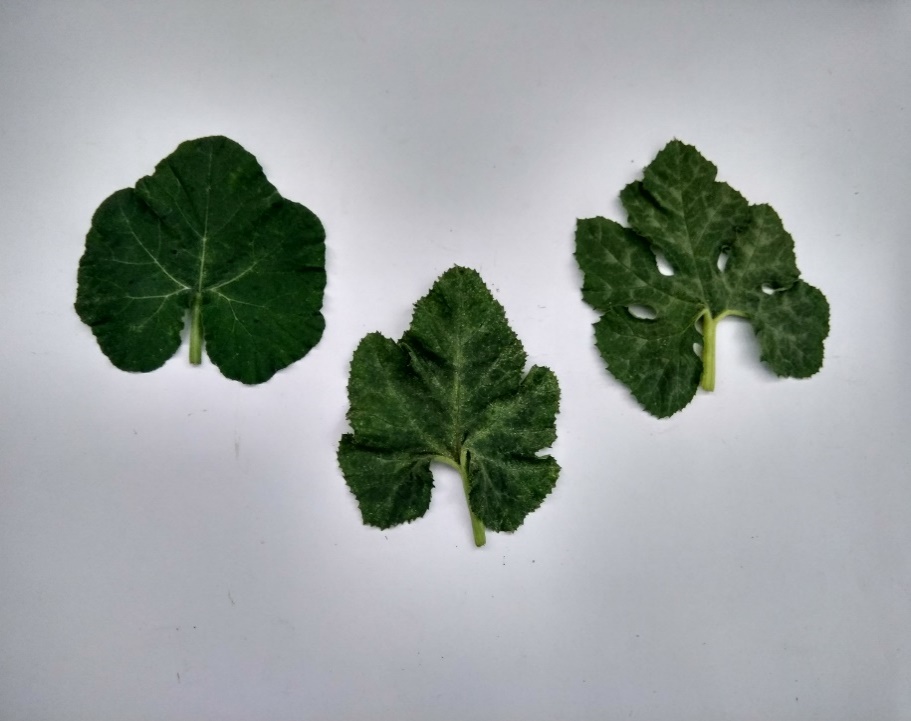**  **G** | | |
| **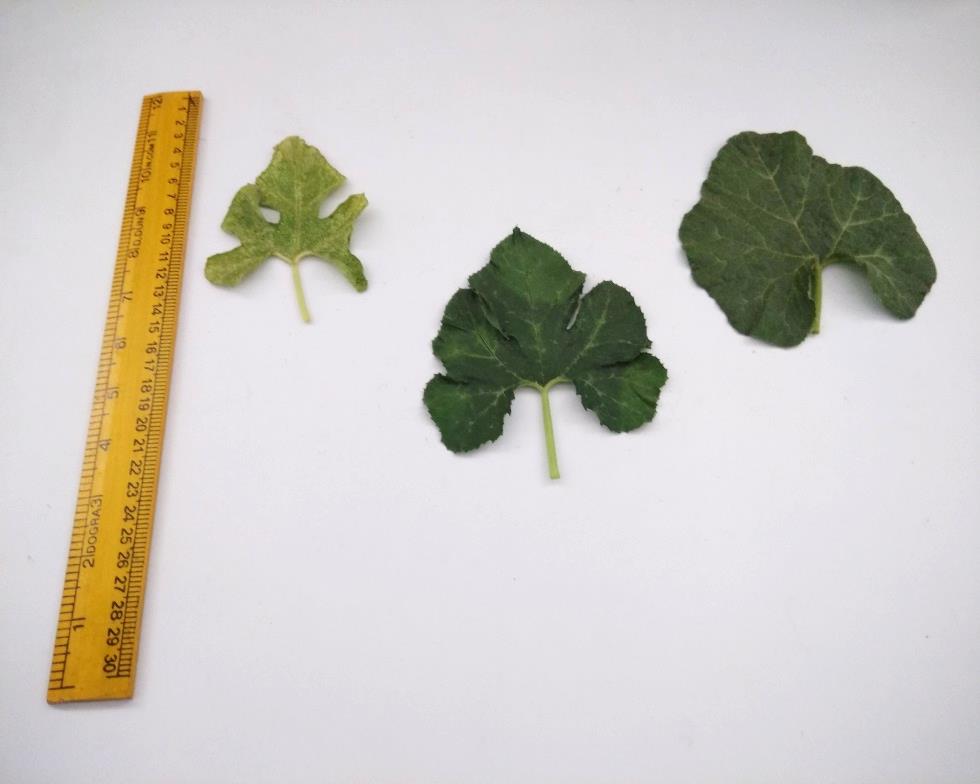**  **H** | **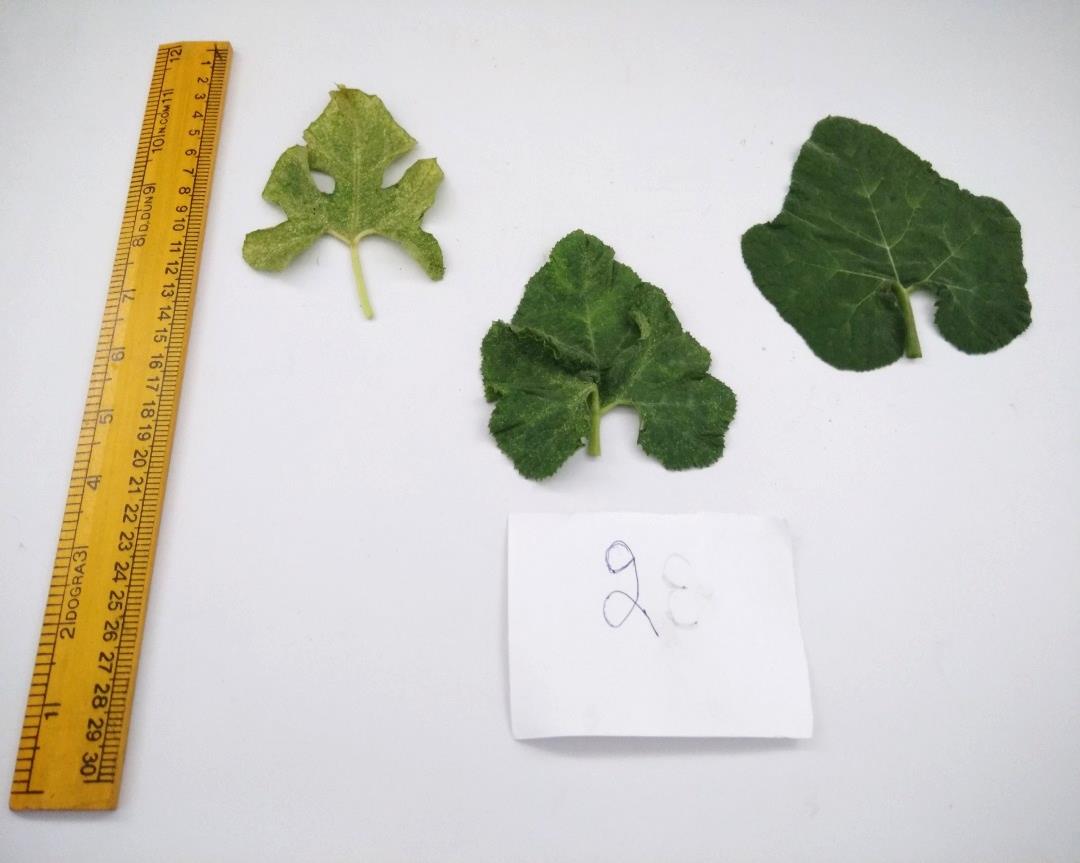**  **I** | | | **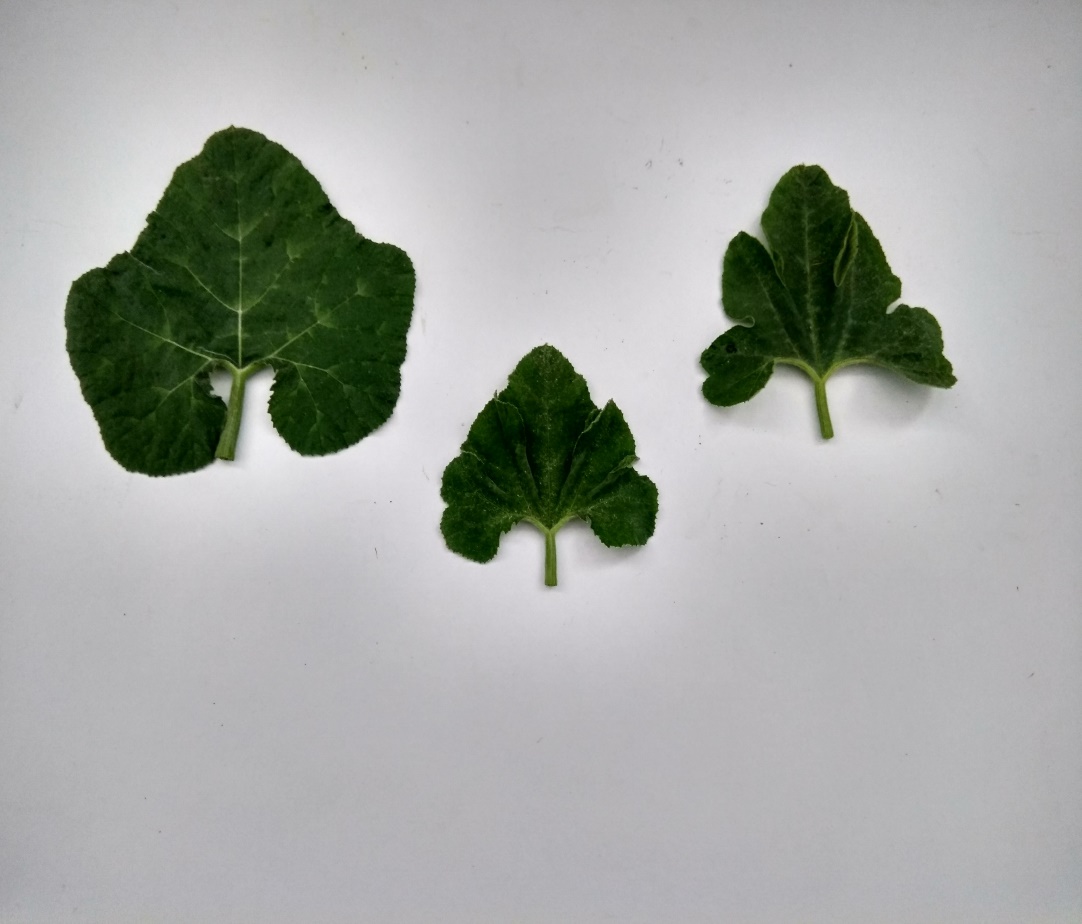**  **J** | | **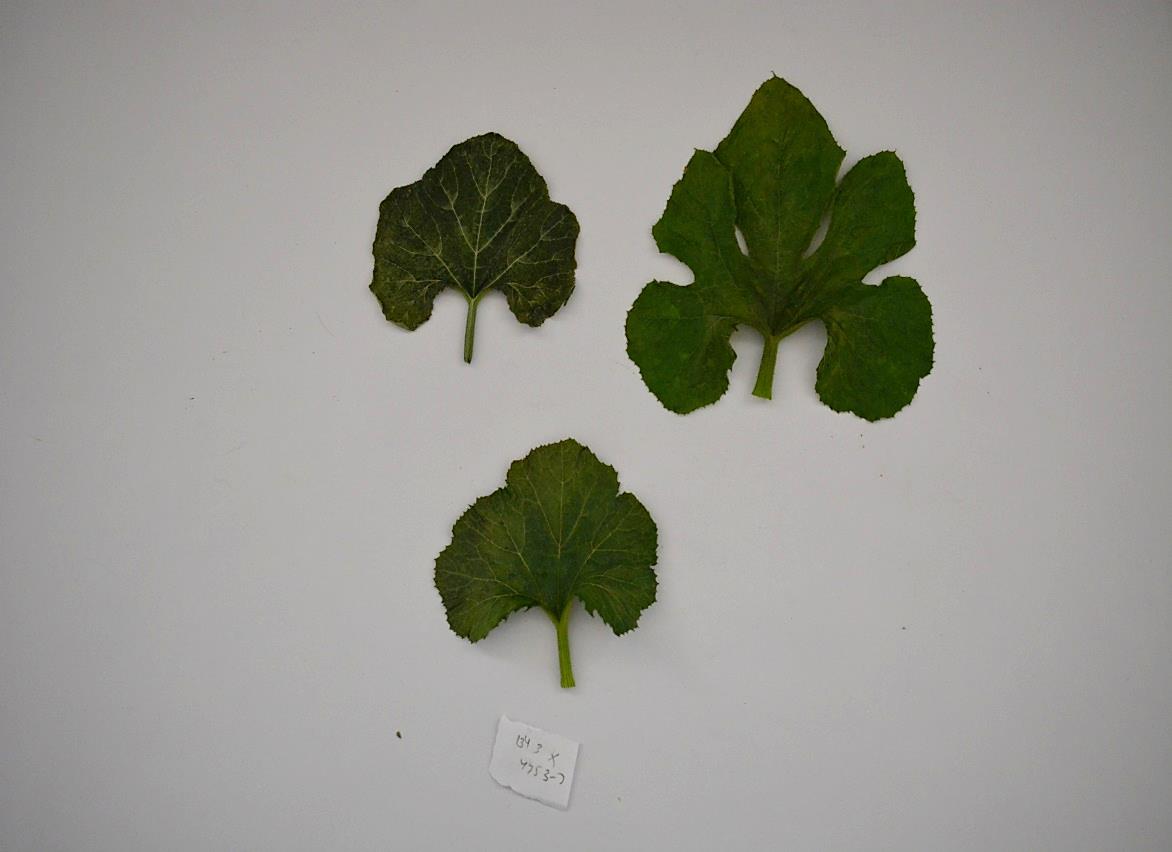**  **K** | | | **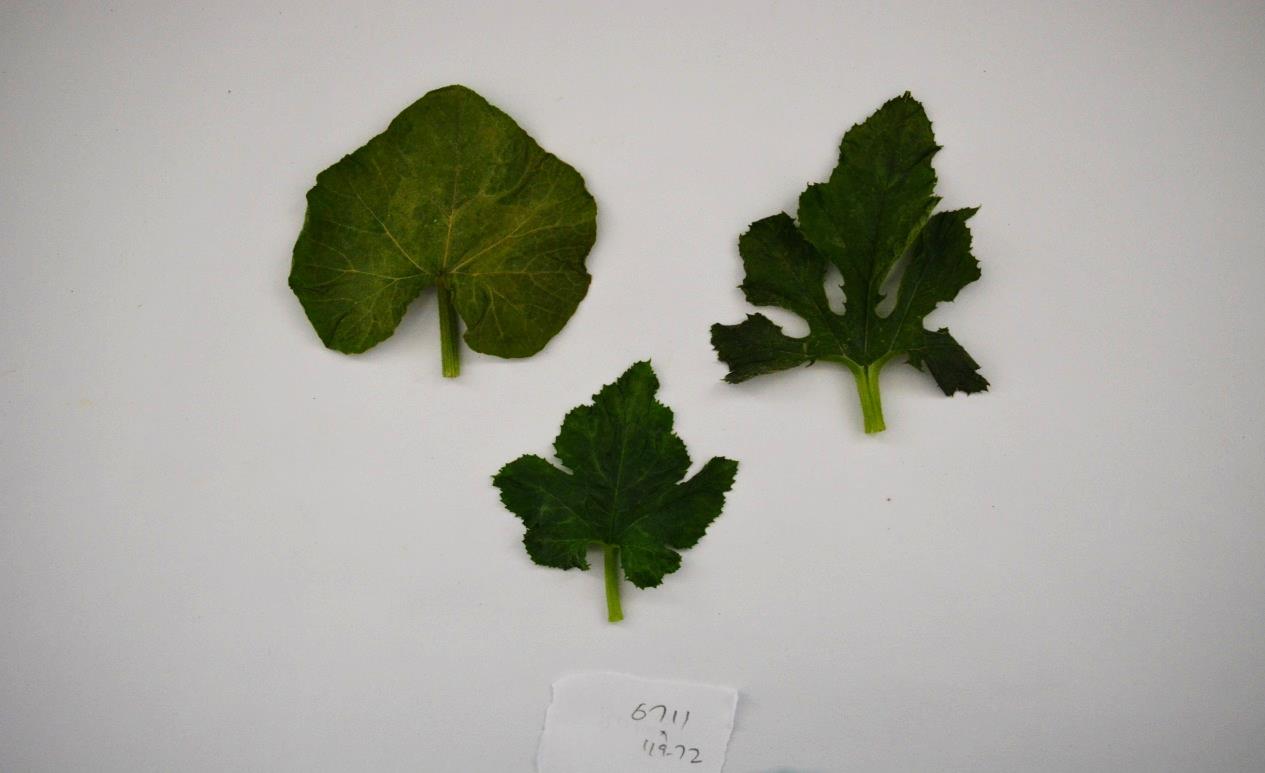**  **L** |

**Supplementary Figure 1. Leaf morphology among parents: *C. pepo*: (A)** HLP36, **(B)** HLP44, **(C)** HLP53, **(D)** HLP72; ***C. moschata*: (E)** HM1343, **(F)** HM1022, **(G)** HM6711; **and interspecific hybrids: (H)** HLP36 × HM1343 (Family I), **(I)** HLP36 × HM1022 (Family II), **(J)** HLP44 × HM1022 (Family III), **(K)** HM1343× HLP53 (Family IV), **(L)** HM6711× HLP72 (Family V).

| **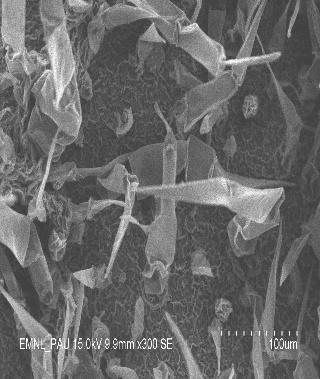**  **A** | | 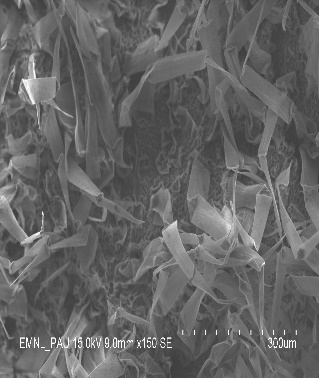  **B** | | | 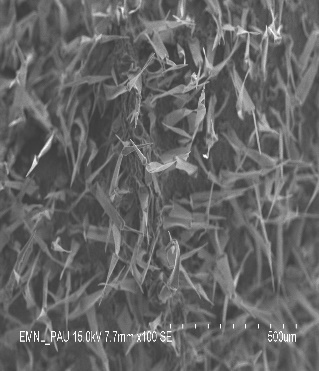  **C** | | | 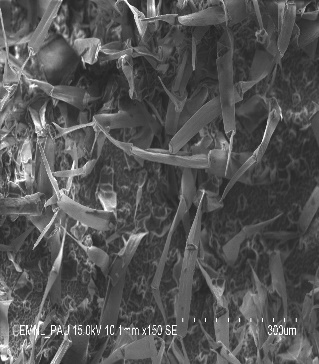  **D** | |
| --- | --- | --- | --- | --- | --- | --- | --- | --- | --- |
| **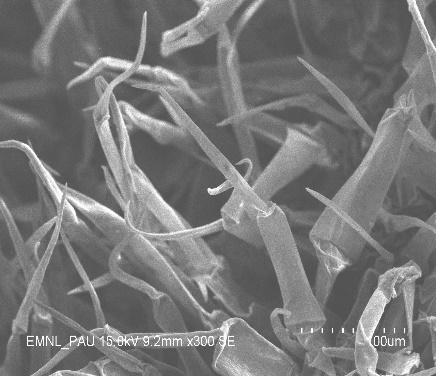**  **E** | | | 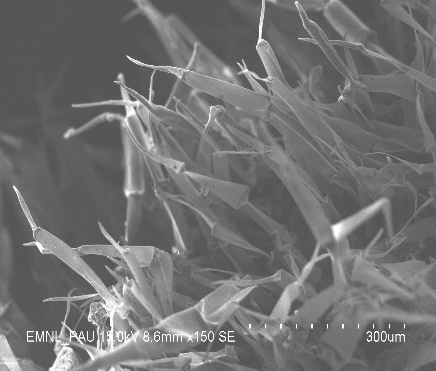  **F** | | | | 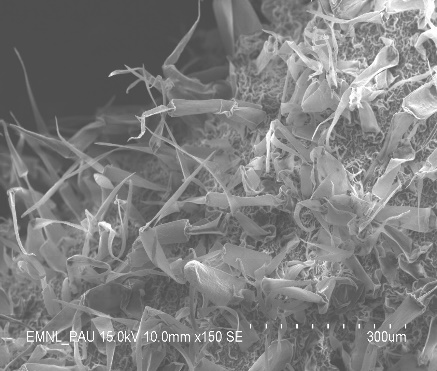  **G** | | |
| **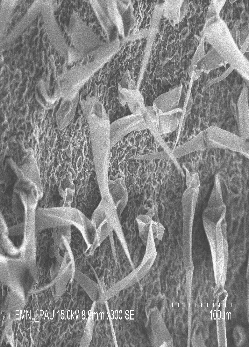**  **H**   1. **B** | 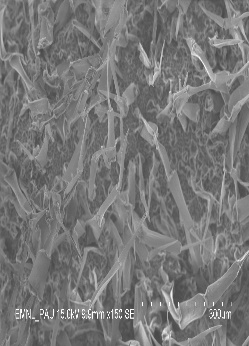  **I** | | | 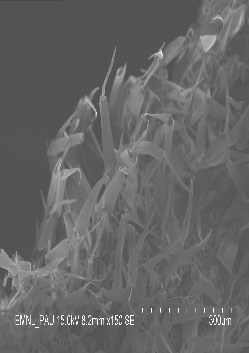  **J** | | 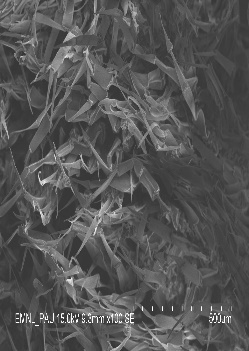  **K** | | | 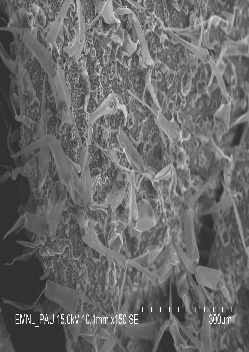  **L** |

**Supplementary Figure 2. Scanning electron micrographs representing trichomes among parents: *C. pepo* (A-D), *C. moschata* (E-G) and interspecific hybrids (H-L).**

**(A)** HLP36: Bar represents 100 μm, **(B)** HLP44: Bar represents 300 μm, **(C)** HLP53: Bar represents 500 μm, **(D)** HLP72: Bar represents 300 μm, **(E)** HM1343: Bar represents 100 μm, **(F)** HM1022: Bar represents 300 μm, **(G)** HM6711: Bar represents 300 μm, **(H)** HLP36 × HM1343 (Family I): Bar represents 100 μm, **(I)** HLP36 × HM1022 (Family II): Bar represents 300 μm, **(J)** HLP44 × HM1022 (Family III): Bar represents 300 μm, **(K)** HM1343× HLP53 (Family IV): Bar represents 500 μm, **(L)** HM6711× HLP72 (Family V): Bar represents 300 μm.

| 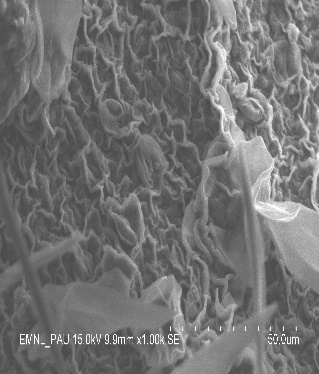  **A** | | 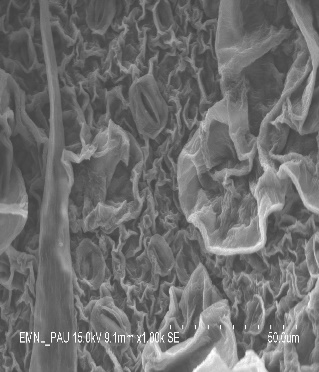  **B** | | | 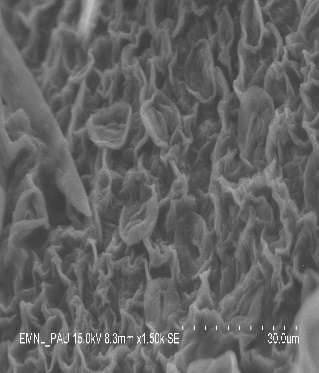  **C** | | | 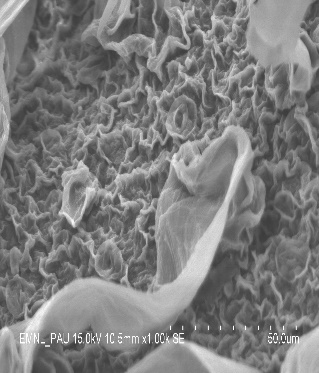  **D** | |
| --- | --- | --- | --- | --- | --- | --- | --- | --- | --- |
| 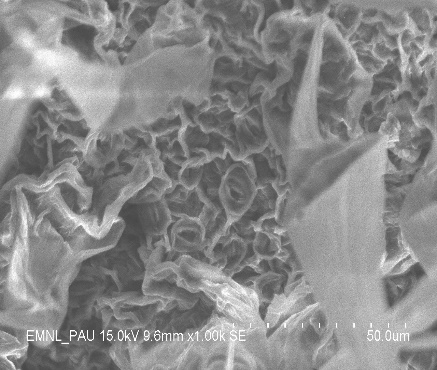  **E** | | | 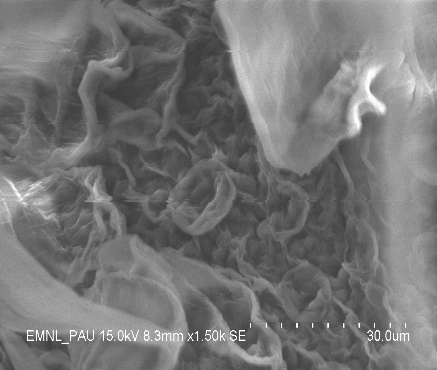  **F** | | | | 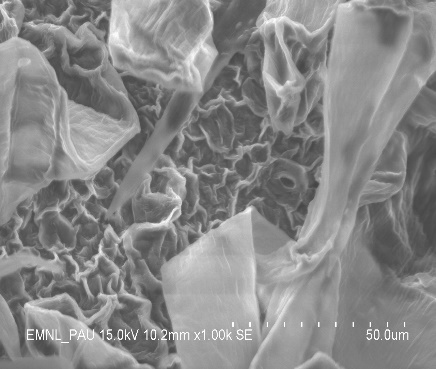  **G** | | |
| 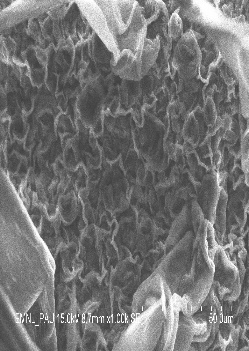  **H** | 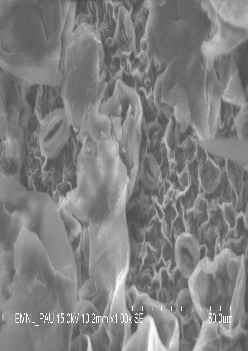  **I** | | | 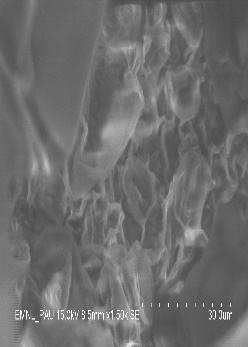  **J** | | 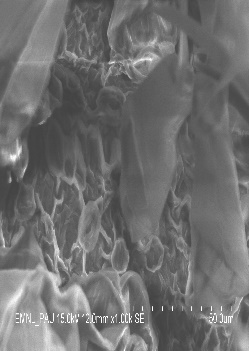  **K** | | | 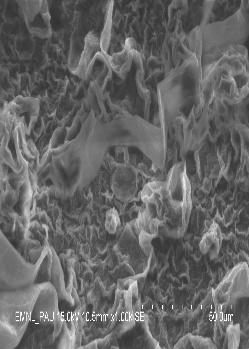  **L** |

**Supplementary Figure 3. Scanning electron micrographs representing stomatal apertures among parents: *C. pepo* (A-D), *C. moschata* (E-G) and interspecific hybrids (H-L).**

**(A)** HLP36: Bar represents 50 μm, **(B)** HLP44: Bar represents 50 μm, **(C)** HLP53: Bar represents 30 μm, **(D)** HLP72: Bar represents 50 μm, **(E)** HM1343: Bar represents 50 μm, **(F)** HM1022: Bar represents 30 μm, **(G)** HM6711: Bar represents 50 μm, **(H)** HLP36 × HM1343 (Family I): Bar represents 50 μm, **(I)** HLP36 × HM1022 (Family II): Bar represents 50 μm, **(J)** HLP44 × HM1022 (Family III): Bar represents 30 μm, **(K)** HM1343 × HLP53 (Family IV): Bar represents 50 μm, **(L)** HM6711× HLP72 (Family V): Bar represents 50 μm.

**
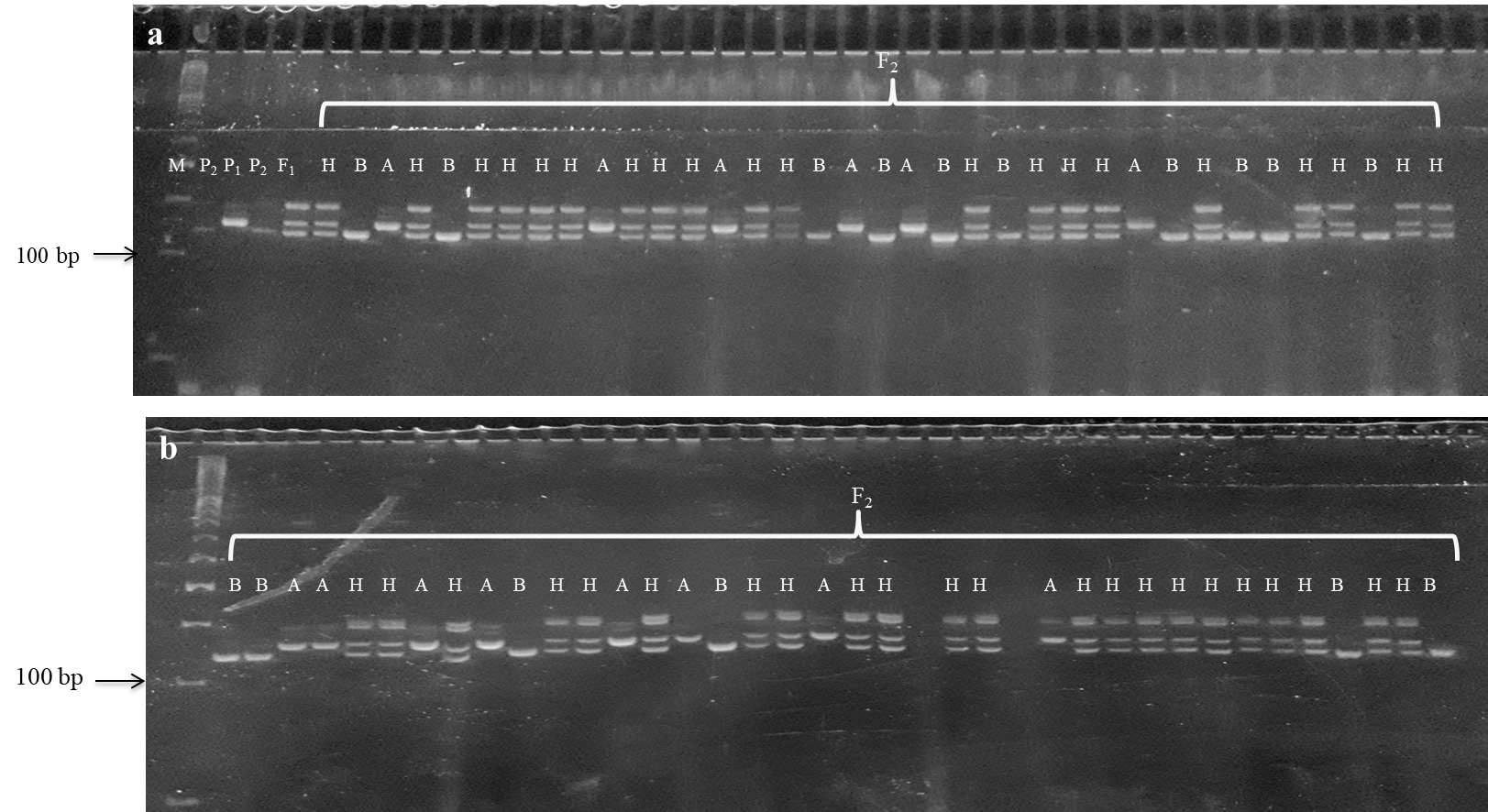
**

**Supplementary Figure 4 (a&b): Genotyping of interspecific F_2_ (HLP36 × HM1343) for hull-less seed trait using SSR marker, CMTp182**

M: DNA ladder (50bp), P_1_: Hull-less parent; P_2_: Hulled parent; F_1_: Interspecific F_1_ hybrid, F_2_: segregating interspecific F_2_ population, A: P_1_ type, B: P_2_ type, H: Heterozygote between A and B
